# Successful Management of a Complex Coronary Bifurcation Lesion in Acute Coronary Syndrome Using a Free‐Metal Approach

**DOI:** 10.1002/ccd.70553

**Published:** 2026-03-12

**Authors:** Flavius‐Alexandru Gherasie, Claudiu Ungureanu

**Affiliations:** ^1^ Emergency Clinical Hospital Dr. Bagdasar‐Arseni Bucharest Romania; ^2^ Cardiovascular Jolimont Hospital La Louvière Belgium

**Keywords:** bifurcation lesion, coronary intervention, cutting balloon angioplasty, drug‐coated balloon, intravascular ultrasound, reverse wire technique, Rodin‐CUT technique, stentless PCI

## Abstract

We present the case of a 56‐year‐old male with non‐ST elevation myocardial infarction (NSTEMI) and a complex thrombotic lesion at the ostial left circumflex artery (LCX), extending into bifurcating marginal branches. Due to the high risk of side branch occlusion, a stentless strategy using thrombus aspiration, the reverse wire technique, and sequential plaque modification with a cutting balloon (Rodin‐CUT technique) was employed. Following optimal lesion preparation, a drug‐coated balloon (DCB) was deployed, achieving excellent angiographic and intravascular ultrasound (IVUS) results, with sustained vessel patency at 6‐month follow‐up. This case highlights the efficacy of a free‐metal strategy in managing challenging bifurcation lesions while preserving side branches and avoiding the risks associated with permanent metallic implants.

## 1 Clinical Presentation

A 56‐year‐old male with a preserved left ventricular ejection fraction underwent coronary angiography following a non‐ST elevation myocardial infarction (NSTEMI). The culprit lesion involved the ostial circumflex artery, with thrombus extension toward the first and second marginal branches (Panel A, Videos S1 and S2). The initial strategy aimed to avoid stenting the bifurcation due to the risk of side branch occlusion. Instead, the plan involved thrombus aspiration followed by drug‐eluting balloon therapy.

## 2 Procedural Challenges

Wiring the LCX was complicated by severe tortuosity, including *a* > 270° angle between the first marginal branch and proximal LCX. Initial attempts to advance the wire distally using an over‐the‐wire (OTW) balloon failed. However, on the second attempt, the vessel was successfully wired using the reverse wire technique [1] (Panels B and C, Videos S3 and S4).

## 3 Lesion Preparation and Treatment

Given the presence of moderate to severe calcifications at the ostial circumflex, lesion preparation was performed using the Rodin‐CUT technique [2]. This technique involved multiple high‐pressure inflations of a 3 mm cutting balloon (Wolverine, Boston Scientific, USA) at various points along the lesion to enhance plaque modification (Panel D, Video S5).

Following successful expansion of a 3.5 mm non‐compliant balloon at low pressure (4 atm), interpreted as an indicator of optimal plaque preparation, a drug‐coated balloon (Prevail DCB, Medtronic, USA) was deployed and inflated at 8 atm for 1 min (Panel E, Video S6).

## 4 Final Outcome

The final angiographic result was excellent, with TIMI 3 flow in all vessels, preservation of side branches, and less than 30% residual stenosis. A minor type A dissection was observed and deemed acceptable in this context [3] (Panel F, Video S7).

## 5 Follow‐Up

At 6‐month follow‐up, angiography and intravascular ultrasound (IVUS) confirmed the durability of the result. IVUS revealed a large lumen area of 8.8 mm² at the ostial circumflex, with disease‐free marginal branches (Panels G and I, Videos S8 and S9).

## 6 Discussion

This particular case, involving the concomitant presence of several challenging anatomical features, including an ostial circumflex lesion, severe calcification, significant thrombotic burden, and bifurcation anatomy, raised important concerns regarding the risks of thrombus embolisation during plaque debulking, side branch occlusion, and stent underexpansion at the coronary ostium.

This case demonstrates the feasibility and advantages of a stentless, free‐metal strategy for managing a complex coronary bifurcation lesion in acute coronary syndrome. The clinical scenario involved a thrombotic ostial LCX lesion with significant calcification and acute angulation toward marginal branches. These anatomical and morphological features increase procedural complexity and elevate the risk of complications such as side branch occlusion, malapposition, or underexpansion when metallic stents are used.

The incremental clinical message of this case does not reside in the introduction of a new device or technique, but in the deliberate sequencing and integration of established tools in a high‐risk anatomical setting.

In a thrombotic ostial LCX bifurcation with severe angulation and calcification, a stepwise approach combining thrombus aspiration, the reverse wire technique, controlled plaque modification using the Rodin‐CUT method, and DCB angioplasty allowed effective lesion treatment while preserving both marginal branches and avoiding permanent metallic implantation. Each step was tailored to the lesion's characteristics to reduce procedural manipulation, minimize thrombus migration, and preserve side branch patency.

The more recent Rodin‐CUT technique, based on multiple high‐pressure inflations of a cutting balloon, enabled effective plaque modification and fissuring as a standalone primary approach, resulting in significant acute luminal gain despite the severity of calcification and without the need for additional debulking techniques. This “stent‐like” angiographic result allowed consideration of a stentless strategy and drug‐coating balloon use, which in this specific setting offered potential advantages by reducing the risks of side branch occlusion and restenosis in an anatomically vulnerable segment.

In particular, for ostial circumflex lesions, where extensive calcification and high plaque resistance may hinder optimal stent expansion and predispose to target lesion failure, aggressive plaque modification using dedicated debulking devices, such as rotational atherectomy followed by DCB angioplasty, has been shown to be an effective strategy [4, 5]. However, in the present case, the significant thrombotic burden raised concerns regarding distal embolisation, making such an approach less favorable.

Although intravascular imaging is strongly recommended in stentless and bifurcation PCI, IVUS was deferred during the index procedure to limit catheter manipulation in the acute thrombotic setting. Nevertheless, its importance in guiding balloon sizing, evaluating plaque modification, and assessing dissections is fully acknowledged. Moreover, the procedural outcome was excellent both angiographically and clinically, and IVUS was performed at 6‐month follow‐up, confirming a stable and patent vessel without late complications.

**Figure 1:**
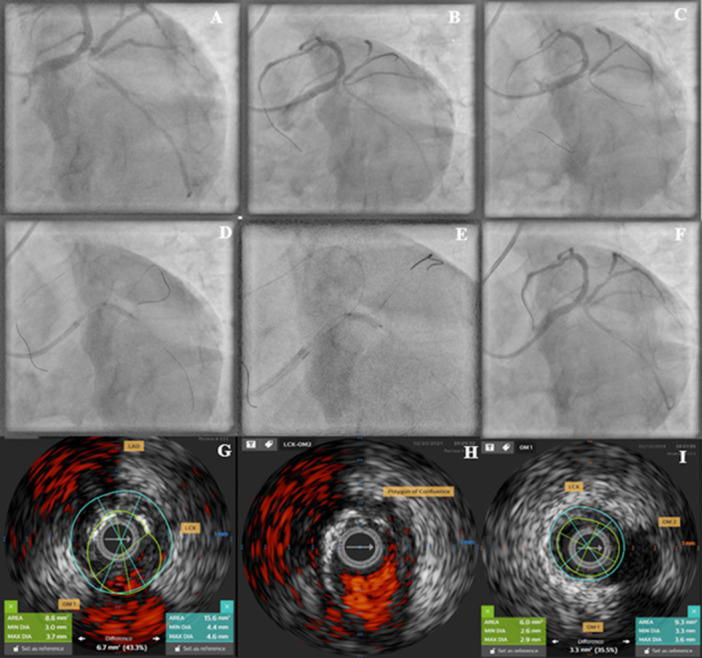
(A) Basal angiography. (B−E) vessel wiring and preparation. (F) Final result. (G−I) IVUS findings at 6 months. (G) ChromaFlow, ostial LCX, good MLA, fibro‐calcified plaque (9−3 o'clock), good OM1 flow, and disease‐free ostial OM1. (H) ChromaFlow, Polygon of Confluence, fibro‐calcified plaque (8‐1 o'clock) in the LCX. (I) Proximal LCX, good LMA, fibrotic plaque, and disease‐free ostial OM2. [Color figure can be viewed at wileyonlinelibrary.com]

Overall, this case highlights the importance of tailoring technical strategies to lesion morphology and clinical context rather than relying on a single standardized approach, in order to achieve procedural success and optimal clinical outcomes (Figure 1).

## Consent

Written informed consent was obtained from the patient to publish this report in accordance with the journal's patient consent policy.

## Conflicts of Interest

The authors declare no conflicts of interest.

## Supporting information


**Video 1.** Basal angiography LAO.

**Video 2.** Basal angiography SPIDER view.

**Video 3.** The first attempt using the reverse wire technique.

**Video 4.** The second attempt using the reverse wire technique.

**Video 5.** Vessel preparation using Cutting Balloon.

**Video 6.** DCB angioplasty.

**Video 7.** Final angiography after DCB angioplasty.

**Video 8.** 6 months angiographic follow‐up LAO.

**Video 9.** 6 months angiographic follow‐up SPIDER view.

## Data Availability

The data to support the findings of this study are available on request from the corresponding author. The data are not publicly available due to privacy or ethical restrictions.
